# Longitudinal ultrasonographic assessment of placentome diameter and maternal ovine placental lactogen across gestation in sheep

**DOI:** 10.3389/fvets.2026.1769704

**Published:** 2026-01-20

**Authors:** Kerem Baykal, Ahmet Sabuncu, Gamze Evkuran Dal, Sinem Özlem Enginler, Aslıhan Baykal Uğur, Merve Yılmaz, Mert Sarılar

**Affiliations:** 1 Institute of Graduate Studies, İstanbul University-Cerrahpaşa, Istanbul, Türkiye; 2 Department of Obstetrics and Gynecology, Faculty of Veterinary Medicine, Istanbul University-Cerrahpaşa, Istanbul, Türkiye

**Keywords:** gestation, placental development, placental lactogen (PL), placentome diameter, sheep, ultrasonography

## Abstract

**Introduction:**

Placental development in sheep involves closely coordinated morphological and endocrine processes. Ultrasonographic placentome measurements and maternal ovine placental lactogen (oPL) profiles reflect distinct components of pregnancy progression; however, their longitudinal relationship across gestation has not been clearly defined. This study aimed to characterize temporal changes in placentome diameter and maternal oPL concentrations and to evaluate how their association evolves throughout pregnancy.

**Methods:**

Fifty clinically healthy, multiparous Kivircik ewes carrying singleton pregnancies were monitored longitudinally from Day 26 to Day 145 of gestation. Serial transabdominal ultrasonography was performed at two-week intervals to measure placentome diameter using consistent anatomical landmarks. Maternal blood samples collected at each examination were analyzed for serum oPL concentrations using validated immunoassays. Gestational changes were assessed using non-parametric repeated-measures analyses. Associations between structural and endocrine variables were examined using day-specific correlation analyses and within-ewe models that accounted for repeated observations and individual baseline variability.

**Results:**

Placentome diameter increased rapidly during early gestation, rising from approximately 1.3 cm at Day 26 to around 3.5 cm by mid-gestation, after which values stabilised and showed only minor fluctuations toward term. Maternal oPL concentrations increased progressively from early pregnancy, reaching peak values of approximately 15–16 μg/mL in late gestation before declining near parturition. No significant association between placentome diameter and oPL concentration was detected during early gestation. Transient inverse relationships were observed at selected mid-gestational time points, whereas from late gestation onward, a consistent positive association emerged. Within-ewe analyses demonstrated a strong positive co-variation between placentome diameter and circulating oPL concentrations after controlling for inter-individual differences.

**Discussion:**

These findings indicate that placentome growth and oPL secretion follow distinct yet interrelated developmental trajectories during sheep gestation, with increasing structural–endocrine concordance in late pregnancy. The integrated longitudinal evaluation of placentome biometry and maternal oPL profiles provides a refined framework for interpreting placental development and may support improved assessment of gestational progression in both research and clinical settings.

## 1 Introduction

Sheep flocks represent a central component of small-ruminant production systems, and the efficiency of these systems is closely associated with the effective management of reproductive performance. Early and reliable detection of pregnancy plays a key role in organizing lambing, reducing prenatal and neonatal losses, and optimizing feeding and husbandry schedules (1–3). For this reason, the reliability of morphometric and endocrine indicators that reflect the physiological progression of gestation is critical for both clinical reproductive management and research-based assessments.

In sheep, the placenta is classified as syndesmochorial type, in which maternal and fetal tissues remain closely contact while remaining histologically distinct. Maternal–fetal exchange of nutrients and metabolic products takes place through placentomes, which form when fetal chorionic villi interlock with the crypts of the maternal caruncular epithelium, generating highly specialized zones that support placental function. In sheep, the fetal component of the placenta arises from the fusion of the chorion with the vascular allantois, producing a chorioallantoic membrane that enlarges its functional surface area through villous projections grouped into cotyledons. Each cotyledon associates with a corresponding maternal caruncle to form a placentome. According to literature knowledge, placentomes exhibit a marked variation in sheep, typically numbering 20 to 70 per fetus and ranging in size from less than 10 mm in early gestation to over 50 mm in late pregnancy. According to literature knowledge, placentomes exhibit a marked variation in sheep, typically numbering 20 to 70 per fetus and ranging in size from less than 10 mm in early gestation to over 50 mm in late pregnancy, with smaller placentomes generally located toward the distal ends of the uterine horns and larger ones positioned closer to the bifurcation region (4–6).

Evaluating fetal growth and developmental progression relies largely on ultrasonographic biometry, which includes measurements of both fetal structures and the placental unit (7). In this context, placentome measurements have been widely investigated as a practical indicator of gestational progression due to their measurable and physiologically regulated growth pattern (7–12). Although studies report some variation in the gestational profile of placentome size, the overall pattern indicates that placentomes enlarge progressively from early gestation to mid-pregnancy, followed by a phase of minimal change (7, 12) or a reduction as parturition approaches (9–11). Taken together, these findings highlight placentome measurement as a valuable component of gestational monitoring in sheep, particularly during early and mid-pregnancy.

Ovine placental lactogen (oPL) is a major placenta-derived hormone produced by chorionic binucleate cells and represents one of the key ruminant members of the growth hormone/prolactin gene family. Although structurally more similar to prolactin than to growth hormone, oPL displays distinct endocrine characteristics and enters the maternal circulation as binucleate cells migrate and fuse with the fetomaternal syncytium (13). Its gene is located in close association with the prolactin gene in sheep, and the hormone itself is a nonglycosylated 23-kDa polypeptide composed of 198 amino acids, secreted into both maternal and fetal circulations as trophoblast development progresses (14).

oPL concentrations increase progressively from early pregnancy and reach a plateau during mid-gestation, a pattern driven primarily by the expansion of the chorionic epithelium and the corresponding rise in the number of hormone-secreting binucleate cells rather than changes in per-cell expression activity (15). Circulating levels later decline as parturition approaches and are positively associated with placental mass and fetal number, reflecting the hormone’s cotyledon-derived origin (14, 15). Physiologically, oPL is regarded as an important mediator of maternal metabolic adaptation, supporting preferential nutrient allocation to the fetus and contributing to anabolic processes essential for fetal growth (13).

Given that placentome growth and oPL secretion represent two core dimensions of placental function, morphological development and endocrine output, their joint assessment offers an opportunity to elucidate how structural and hormonal processes progress in parallel throughout gestation. Despite the availability of separate lines of research on placentome growth and oPL secretion, the physiological relationship between these two processes has not been systematically examined throughout gestation. This doctoral research was therefore designed to determine whether placentome diameter and maternal oPL concentrations reflect complementary facets of a shared physiological pattern and to describe how their association evolves from early to late gestation. In this context, serial ultrasonographic placentome measurements and corresponding serum oPL analyses were obtained in Kivircik ewes between Days 26 and 145 of gestation. By evaluating these indicators within the same longitudinal framework, this study aimed to generate a clearer depiction of placental dynamics in sheep and to provide an updated, breed-specific reference for interpreting the relationship between placental growth and endocrine activity.

## 2 Materials and methods

### 2.1 Determination of sample size

A statistical power analysis was conducted to determine the required number of sample size. An *a priori* calculation performed using G*Power 3.1.9.7 for a simple linear regression model (slope-based) assumed an effect size of 0.35, an alpha level of 0.05, and a statistical power of 80%. The analysis indicated that at least 46 ewes would be necessary; therefore, 50 animals were included to account for potential losses during the study period.

### 2.2 Animals and study design

The study was carried out on a commercial sheep farm located in Istanbul (41°0′49.82″N, 28°56′58.78″E) during the breeding season, covering the period from synchronized breeding initiated in September through late gestation (late January- early February). The flock was maintained under uniform management and environmental conditions and managed semi-intensively with a nutritionally balanced complete diet, with free access to water and mineral salt. Because the animals were continuously kept under these stable conditions, no additional acclimation period was required.

### 2.3 Clinical and reproductive evaluation

Before enrolment, all ewes underwent a comprehensive clinical and reproductive examination, including general health assessment, body condition scoring (BCS) according to Russel et al. (16), udder evaluation, and ultrasonographic reproductive screening to ensure the absence of pathological findings. Clinically healthy multiparous Kivircik ewes aged 2–4 years and having a BCS of 3–4 were considered eligible for further evaluation.

### 2.4 Estrus synchronization and breeding management

Estrus synchronization was routinely implemented by the farm veterinarian as part of the herd reproductive management program. Intravaginal sponges containing 60 mg medroxyprogesterone acetate (Esponjavet, Hipra, Spain) were inserted for 11 days, followed by administration of 400 IU eCG intramuscularly at sponge removal (Oviser, Hipra, Spain). Controlled breeding (Day 0) was performed 48 h later using six fertility-verified rams whose ejaculates exhibited 40–60% progressive motility.

### 2.5 Pregnancy confirmation and selection of the study cohort

Pregnancy diagnosis was performed on Day 26 via transrectal ultrasonographic examination (Esaote Pie Medical MyLab Five Vet, Genoa, Italy). Ewes confirmed to be carrying a viable singleton pregnancy and fulfilling all predefined clinical selection criteria were enrolled. This procedure resulted in the establishment of the final study cohort, consisting of 50 ewes.

### 2.6 Ultrasonographic examinations and placental measurements

Ultrasonographic examinations were conducted at predetermined two-week intervals from Day 26 (D26) to Day 145 (D145), enabling systematic monitoring across early, mid-, and late gestation.

On D26, examinations were performed transrectally to ensure reliable confirmation of singleton pregnancies. From D33 onward, a transabdominal approach was adopted to minimize handling-related stress while maintaining adequate image quality. All scans were performed with the ewes in a standing position. A 6.6-MHz linear transducer was used for the initial transrectal assessment, whereas subsequent abdominal scans were conducted with a 5-MHz micro-convex probe. When transabdominal visualization was insufficient, the transrectal method was repeated. The general approach aligns with previously described protocols for ovine reproductive ultrasonography (17–19).

**Figure 1:**
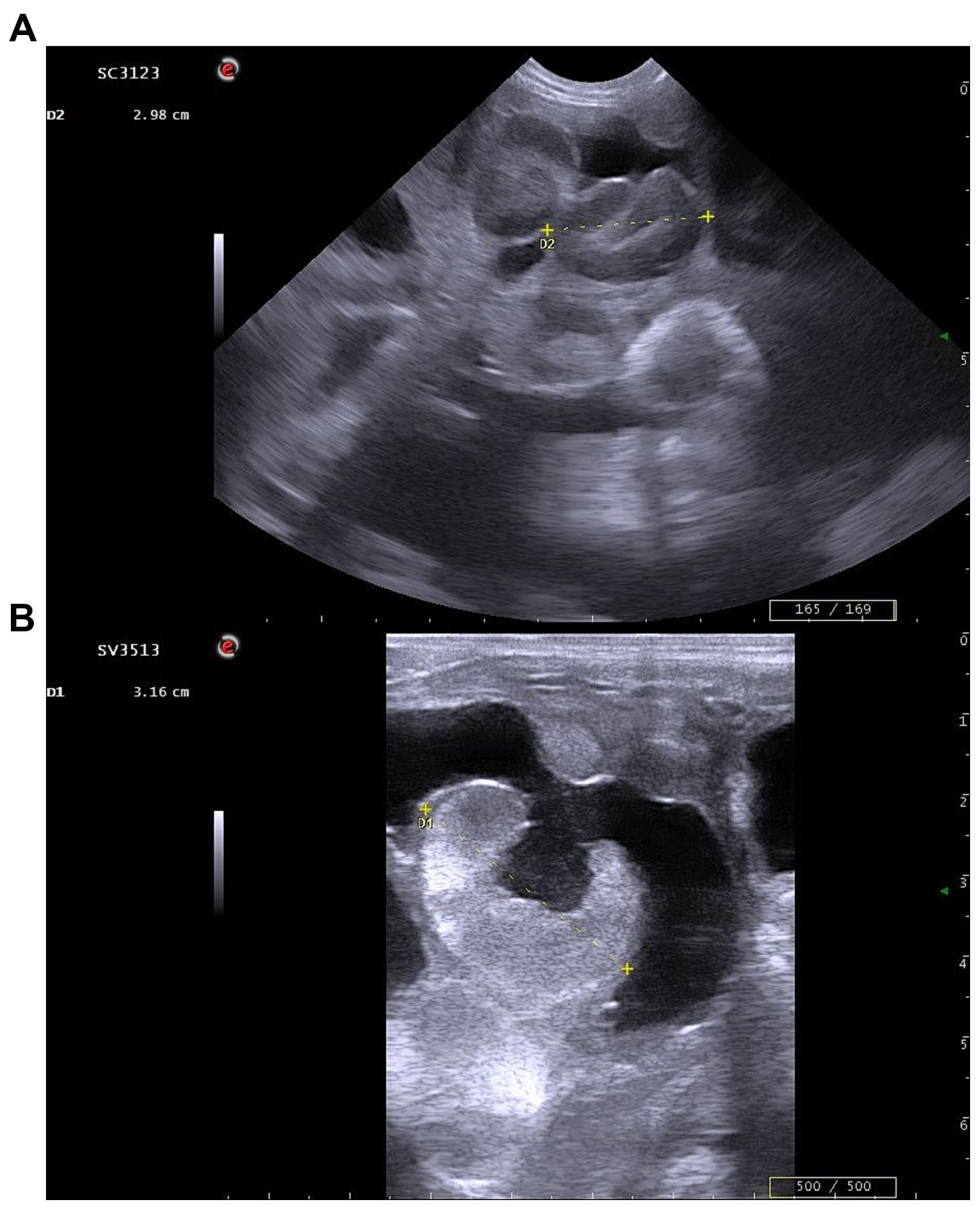
Representative ultrasonographic images illustrating placentome diameter measurements. **(A)** Transabdominal imaging at D40 of gestation; **(B)** Transrectal imaging at D54 of gestation. Images represent examples of placentome diameter measurements, defined as the maximal cross-sectional distance assessed using real-time B-mode ultrasonography.

During each examination, both uterine horns were systematically scanned, and placentomes were evaluated with consideration of their characteristic anatomical distribution. As noted by Alexander (4), the largest placentomes tend to be located near the uterine bifurcation rather than at the distal portions of the horns; therefore, measurements focused on this region to ensure consistent identification of the most representative structures. For each time point, the three largest placentomes visualized were measured, and their mean value was recorded as the placentome diameter (Figure 1).

This approach was selected to reduce measurement variability and provide a standardized biometric indicator, as recommended by Vannucchi et al. (7). Fetal viability was also assessed during each examination.

### 2.7 Blood sampling and oPL analysis

At each ultrasonographic examination, blood samples were collected from the jugular vein using sterile vacuum tubes without anticoagulant. Samples were allowed to clot at room temperature and were transported to the laboratory under cold-chain conditions to minimize degradation. Serum was separated by centrifugation in a refrigerated centrifuge (1,200 × g, 10 min) and aliquots were stored at −40 °C until analysis.

Maternal serum oPL concentrations were quantified using a commercially available ELISA kit validated for ovine samples (BT LAB, China; lot no: 202010007), following the manufacturer’s instructions. The assay sensitivity was 0.043 μg/mL with a dynamic range up to 30 μg/mL. In total, 600 serum samples obtained from 50 ewes across 12 time points were analyzed.

### 2.8 Statistical analysis

For each examination day, the mean placentome diameter was derived from the three largest placentomes measured. Descriptive statistics (mean ± SD, coefficient of variation) were calculated to characterise temporal patterns in placentome size and maternal oPL concentrations. Because measurements were obtained repeatedly from the same animals, overall changes across gestation were analyzed using the non-parametric Friedman test, with effect size reported as Kendall’s W. When a significant time effect was identified, pairwise comparisons between consecutive examination days (e.g., D26–D33, D33–D40, …, D138–D145) were performed using the Wilcoxon signed-rank test, and Holm’s step-down procedure was applied to control the family-wise error rate. The day-specific relationship between placentome diameter and oPL concentration was evaluated using Pearson’s correlation coefficient for each examination day. To account for inter-individual baseline differences, within-ewe correlations were also computed. For this purpose, each ewe’s placentome and oPL values were centred around its individual mean, and Pearson correlations were calculated using these deviation scores to evaluate within-subject co-variation. All tests were two-tailed, and statistical significance was set at *α* = 0.05.

## 3 Results

### 3.1 Temporal changes in placentome diameter and oPL concentration

Both placentome diameter and maternal oPL concentrations showed clear temporal variation across gestation. Descriptive data for all examination days are presented in Table 1.

**Table 1:** Maternal oPL concentrations (μg/mL) and placentome diameter (cm) according to gestational days.

Day	oPL mean ± SD (μg/mL)	oPL CV (%)	Placentome mean ± SD (cm)	Placentome CV (%)
D26	5.64 ± 1.86	32.96	1.26 ± 0.53	42.49
D33	5.94 ± 1.80	30.36	1.86 ± 0.65	34.77
D40	7.52 ± 2.03	27.03	2.40 ± 0.65	27.25
D54	8.16 ± 2.53	31.07	3.55 ± 0.63	17.71
D68	9.19 ± 2.66	29.01	3.54 ± 0.57	16.18
D82	10.02 ± 2.89	28.87	3.66 ± 0.51	13.90
D96	11.11 ± 3.45	31.06	3.54 ± 0.58	16.33
D110	12.64 ± 3.82	30.22	3.69 ± 0.58	15.67
D124	13.92 ± 4.66	33.45	3.47 ± 0.60	17.22
D131	14.96 ± 4.86	32.45	4.11 ± 0.59	14.39
D138	15.60 ± 5.32	34.11	4.21 ± 0.68	16.10
D145	13.26 ± 4.71	35.54	3.86 ± 0.53	13.85

Placentome diameter increased markedly from early pregnancy onward, rising from 1.26 ± 0.53 cm at D26 to 3.55 ± 0.63 cm at D54. During this early period, the range of individual values was wide (e.g., 0.11–2.91 cm at D26), indicating substantial inter-ewe variability despite the overall upward trend. During mid-gestation (D68–D110), placentome size remained relatively stable, with mean values fluctuating between 3.54 and 3.69 cm. The largest placentome diameters were recorded on D131 (4.11 ± 0.59 cm) and D138 (4.21 ± 0.68 cm), followed by a modest decrease at D145 (3.86 ± 0.53 cm).

**Figure 2:**
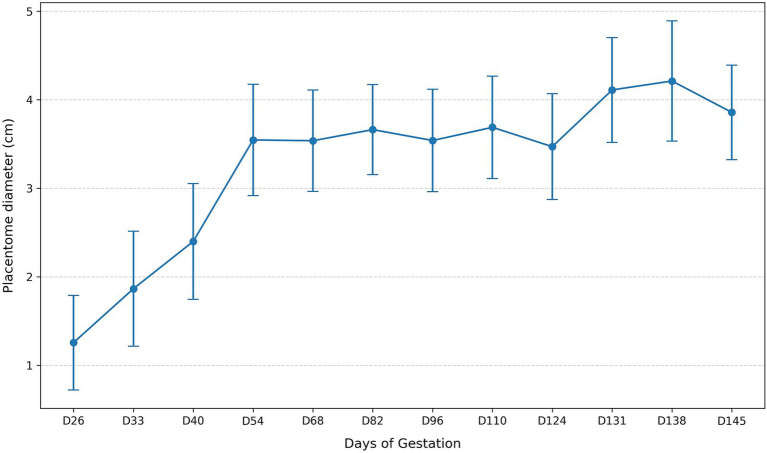
Temporal changes in mean placentome diameter across gestation.

The Friedman test confirmed a significant overall effect of gestational day on placentome diameter (χ^2^(11) = 344.47, *p* < 0.001; Kendall’s W = 0.626). Pairwise comparisons between consecutive gestational time points (Table 2), performed using the Wilcoxon signed-rank test with Holm correction for multiple testing, revealed significant increases during early gestation (D26–D33, D33–D40, D40–D54), whereas most intervals between D54 and D124 were not statistically different (Table 2). A further significant increase occurred from D124 to D131, and a significant decline was detected between D138 and D145. The temporal pattern of placentome growth is illustrated in Figure 2.

**Table 2:** Pairwise comparisons of consecutive days for placentome diameter.

Comparison	*p* (unadjusted)	*p* (holm-adjusted)
D26 vs. D33	9.52 × 10^−13^ (*p* < 0.001)	9.52 × 10^−12^ (*p* < 0.001)
D33 vs. D40	5.32 × 10^−10^ (*p* < 0.001)	4.25 × 10^−9^ (*p* < 0.001)
D40 vs. D54	1.56 × 10^−13^ (*p* < 0.001)	1.72 × 10^−12^ (*p* < 0.001)
D54 vs. D68	0.7961	0.7961
D68 vs. D82	0.0419	0.2097
D82 vs. D96	0.2080	0.4159
D96 vs. D110	0.1095	0.4044
D110 vs. D124	0.0189	0.1133
D124 vs. D131	1.44 × 10^−10^ (*p* < 0.001)	1.30 × 10^−9^ (*p* < 0.001)
D131 vs. D138	0.1011	0.4044
D138 vs. D145	4.24 × 10^−8^ (*p* < 0.001)	2.97 × 10^−7^ (*p* < 0.001)

**Figure 3:**
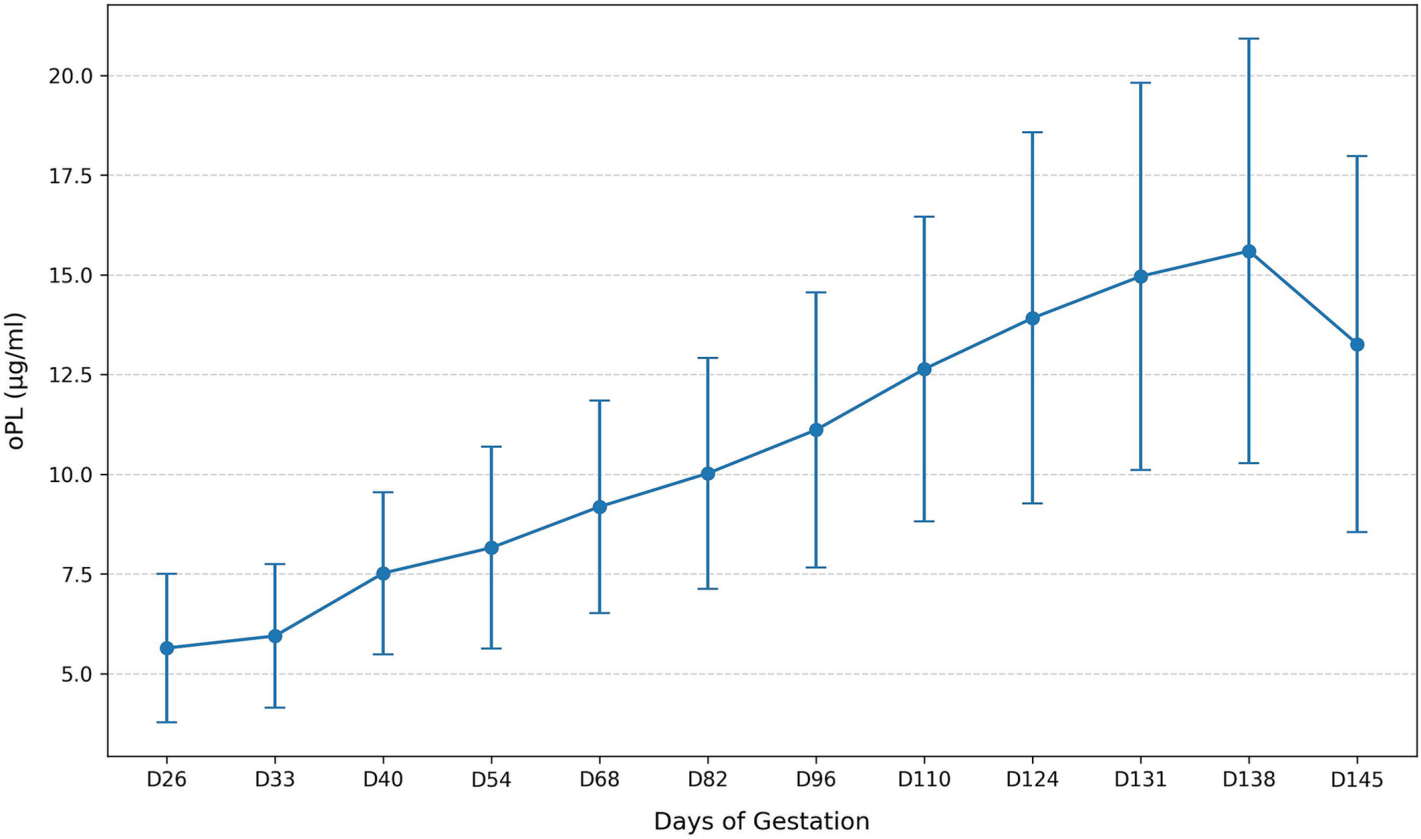
Temporal changes in mean oPL concentrations across gestation.

Maternal oPL concentrations followed a pronounced gestational trajectory, increasing from 5.64 ± 1.86 μg/mL at D26 to 15.60 ± 5.32 μg/mL at D138, with a subsequent decrease to 13.26 ± 4.71 μg/mL at D145 (Table 1). The temporal pattern of oPL concentrations throughout gestation is illustrated in Figure 3. Similar to placentome diameter, early pregnancy oPL values showed broad dispersion (e.g., 2.91–10.76 μg/mL at D26), narrowing progressively as gestation advanced. The Friedman test showed a highly significant overall time effect (χ^2^(11) = 455.21, *p* < 0.001; Kendall’s W = 0.828). Consecutive-day comparisons (Table 3) demonstrated that oPL increased significantly across nearly all intervals, except D131–D138, where the rise was not significant, and D26–D33, where the change was smaller though still significant after correction. The decline between D138 and D145 was also statistically significant.

**Table 3:** Pairwise comparisons of consecutive days for oPL.

Comparison	*p* (unadjusted)	*p* (holm-adjusted)
D26 vs. D33	0.0060	0.0121
D33 vs. D40	3.61 × 10^−12^ (*p* < 0.001)	2.89 × 10^−11^ (*p* < 0.001)
D40 vs. D54	7.16 × 10^−5^ (*p* < 0.001)	0.0002 (*p* < 0.001)
D54 vs. D68	7.64 × 10^−14^ (*p* < 0.001)	8.40 × 10^−13^ (*p* < 0.001)
D68 vs. D82	9.05 × 10^−8^ (*p* < 0.001)	5.43 × 10^−7^ (*p* < 0.001)
D82 vs. D96	4.57 × 10^−7^ (*p* < 0.001)	2.28 × 10^−6^ (*p* < 0.001)
D96 vs. D110	1.95 × 10^−13^ (*p* < 0.001)	1.95 × 10^−12^ (*p* < 0.001)
D110 vs. D124	1.44 × 10^−10^ (*p* < 0.001)	1.01 × 10^−9^ (*p* < 0.001)
D124 vs. D131	9.90 × 10^−6^ (*p* < 0.001)	3.96 × 10^−5^ (*p* < 0.001)
D131 vs. D138	0.0100	0.0121
D138 vs. D145	4.49 × 10^−13^ (*p* < 0.001)	4.04 × 10^−12^ (*p* < 0.001)

Taken together, these findings show that placentome diameter and oPL concentration both vary significantly with gestational progression; however, placentome diameter displays a mid-gestation plateau, whereas oPL rises more continuously until late gestation before declining near term.

### 3.2 Day-specific association between placentome diameter and oPL concentration

**Figure 4:**
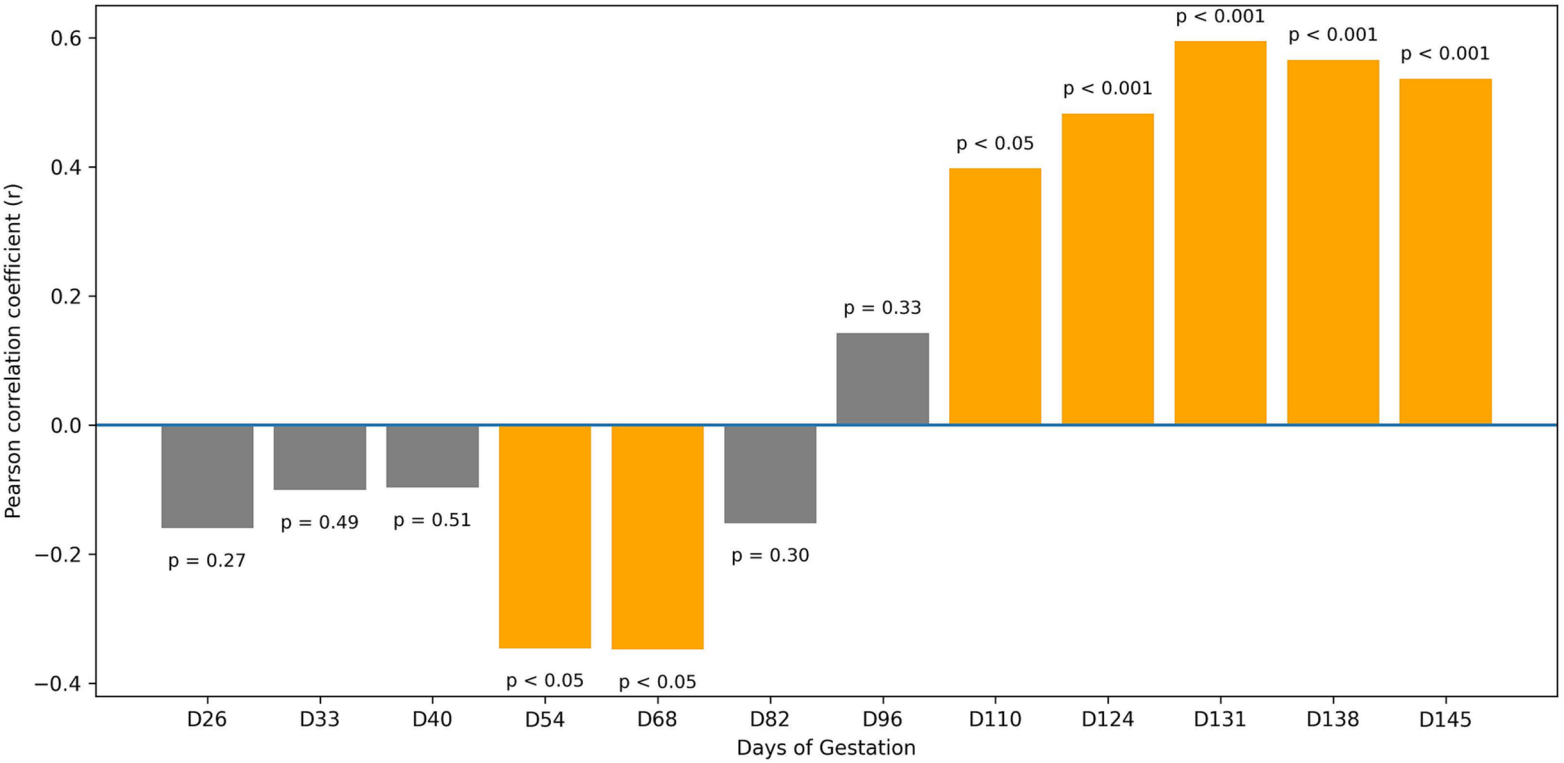
Correlation between oPL and placentome diameter according to gestational days.

The association between placentome diameter and maternal oPL concentration exhibited a time-dependent pattern over the course of gestation (Figure 4). During early pregnancy (D26–D40), no significant correlation was detected between the two variables. Correlation coefficients remained low and non-significant at D26 (r = −0.158, *p* = 0.274), D33 (r = −0.099, *p* = 0.492) and D40 (r = −0.095, *p* = 0.511), consistent with the marked inter-individual variability observed in both measurements during this period.

In mid-gestation, a temporary shift toward an inverse association was observed. Significant negative correlations were recorded on D54 (r = −0.344, *p* = 0.015) and D68 (r = −0.346, *p* = 0.014). On D82, the correlation remained negative but was not statistically significant (r = −0.150, *p* = 0.299).

From D96 onward, correlation coefficients increased progressively. Although the association at D96 was not significant (r = 0.141, *p* = 0.329), significant positive correlations emerged from D110 onwards. Correlation values were r = 0.396 (*p* = 0.004) at D110, r = 0.481 (*p* < 0.001) at D124, r = 0.593 (*p* < 0.001) at D131, r = 0.564 (*p* < 0.001) at D138 and r = 0.535 (*p* < 0.001) at D145 (Table 4).

**Table 4:** Day-specific correlations between oPL and placentome diameter.

Day	Pearson r	*p*
D26	−0.158	0.2741
D33	−0.099	0.4922
D40	−0.095	0.5112
D54	−0.344	0.0145
D68	−0.346	0.0139
D82	−0.150	0.2992
D96	0.141	0.3287
D110	0.396	0.0044
D124	0.481	0.0004 (*p* < 0.001)
D131	0.593	5.67 × 10^−6^ (*p* < 0.001)
D138	0.564	2.00 × 10^−5^ (*p* < 0.001)
D145	0.535	6.37 × 10^−5^ (*p* < 0.001)

### 3.3 Intra-individual association between placentome diameter and oPL concentration across gestation

To account for the repeated-measures structure of the data, placentome diameter and oPL values for each ewe were centered around the individual animal’s own mean (within-subject centering), and correlations were calculated using these deviations. The resulting within-ewe correlation, calculated across all gestational time points, was r = 0.674, indicating a strong synchronous association between placentome diameter and oPL concentration. Accordingly, after removing between-animal differences in absolute baseline levels, increases in placentome diameter within an individual ewe were consistently associated with a corresponding tendency for increased circulating oPL.

## 4 Discussion

Placentome morphology and placental lactogen secretion represent two fundamental aspects of placental function in sheep, and understanding how these processes evolve throughout gestation provides important insight into fetomaternal physiology. Ultrasonographic evaluation of placentomes has long been used to characterise placental growth, with studies consistently demonstrating their progressive enlargement from early pregnancy through mid-gestation (7, 8). oPL, produced by chorionic binucleate cells, follows a well-defined secretory profile that reflects placental development and maternal metabolic adaptation, with circulating concentrations rising from early pregnancy and peaking near late gestation (14, 15). Despite detailed descriptions of each parameter individually, their joint temporal behavior and stage-specific association have not been comprehensively examined. The present study therefore provides an integrated longitudinal assessment of placentome diameter and maternal oPL concentrations in Kivircik ewes, offering a complementary perspective on structural and endocrine aspects of placental maturation.

The temporal pattern of placentome growth observed here aligns with earlier reports describing rapid expansion during early pregnancy, followed by a period of limited change around mid-gestation (7, 8). Although some studies have noted a gradual decline in placentome size as parturition approaches (9–11), the present findings indicate that placentome diameter remained stable through mid-to-late gestation and increased again shortly before term, with only a slight decrease observed at the final examination day. This variability in late-gestation trends mirrors the substantial biological diversity previously documented in placentome morphology. Doize et al. (8) emphasised that placentomes differ not only between individuals but also between uterine horns within the same ewe, owing to asymmetry in caruncular distribution and functional capacity. Such inherent morphological variation, together with breed-specific patterns reported in Enginler et al. (9) and Koldaş Ürer et al. (10), likely contributes to the wide measurement ranges and the modest fluctuations detected in the present study. From a physiological perspective, the apparent stabilisation of placentome diameter during mid-to-late gestation likely reflects placental maturation rather than a cessation of growth. As gestation advances, placentomal development is increasingly characterised by structural remodelling, including enhanced vascularisation, tissue differentiation, and expansion of the maternal–fetal exchange interface-processes that may not be fully captured by linear diameter measurements alone (4, 8, 14). Moreover, progressive changes in placentome shape and thickness during late gestation may further limit the sensitivity of diameter-based assessments despite ongoing functional adaptation of the placenta (6). In this context, the progressive functional maturation of the placentome during mid-to-late gestation may help explain the evolving association observed between placentome diameter and maternal ovine placental lactogen concentrations, reflecting an increasing coordination between placental structure and endocrine activity as pregnancy advances.

Maternal oPL concentrations followed a pattern consistent with established physiological expectations: a progressive rise from early pregnancy through late gestation, reaching near-maximum levels around Days 120–140 before declining toward term, as previously described (14, 15). The marked increase in circulating oPL during mid-gestation corresponds to the expansion of chorionic epithelium and the proliferation of binucleate cells, rather than per-cell transcriptional changes (15). Most available data on placental hormones are derived from cross-sectional or experimental studies, whereas longitudinal observations across gestation remain limited. In this context, evaluating oPL dynamics together with structural placental parameters allows classical endocrine knowledge to be revisited within an integrated and contemporary physiological context. The progressive narrowing of inter-individual variability observed in later gestation in the present study is consistent with the review by Gootwine (14), who highlighted the strong link between placental mass, cotyledonary development and maternal oPL output. Furthermore, nutritional modulation of placental growth illustrates how endocrine activity remains tightly coupled to placental structural capacity, reinforcing the value of assessing these parameters in parallel. Taken together, the present findings extend existing knowledge by demonstrating how the association between placentome growth and maternal oPL concentration evolves in a stage-dependent manner across gestation within a longitudinal framework.

Comparison of temporal profiles revealed that placentome diameter and oPL concentration, although both increasing throughout gestation, do not progress in a fully synchronised manner during early and mid-pregnancy. The absence of a significant correlation in early gestation likely reflects the pronounced biological heterogeneity inherent to initial placentome development, a stage characterised by rapid tissue differentiation, uneven establishment of vascular networks, and marked variability in placentome size and distribution. During this period, oPL secretion may precede or exhibit temporal dissociation from measurable structural growth, as endocrine activity is primarily driven by the functional maturation of trophoblastic cell populations rather than gross placentome dimensions. The transient inverse correlations observed at mid-gestation (days 54 and 68) further suggest that placental growth enters a transitional phase in which structural expansion, vascular remodelling, and endocrine output are not yet temporally aligned. At this stage, short-term differences in placentome perfusion, binucleate cell turnover, and cotyledonary tissue remodelling may differentially influence diameter measurements and oPL release, resulting in temporary divergence between structural and hormonal indices. Importantly, this mid-gestational dissociation appears to represent a physiological reorganisation phase rather than impaired placental function, setting the stage for the tighter structural–endocrine coordination observed in late gestation. Consistent with this interpretation, previous studies have noted that placentome dimensions alone do not necessarily parallel functional or endocrine indicators of gestational progression toward term (9, 12).

A notable finding of the present study is the emergence of a consistent positive correlation from Day 110 onward, indicating increasing alignment between structural and endocrine aspects of placental function in late pregnancy. This observation is compatible with the physiological framework outlined by Gootwine (14), who suggested that oPL secretion becomes more tightly linked to placental mass and cotyledonary development as gestation advances. The strong intra-individual association demonstrated here further supports this interpretation: when between-animal differences were removed, increases in placentome diameter were systematically accompanied by elevations in maternal oPL concentration. This synchrony underscores that, despite variability in absolute levels, structural and hormonal trajectories follow a coordinated pattern within individual ewes.

The final weeks of gestation were characterized by slight decreases in both placentome diameter and oPL concentrations, consistent with previous ultrasonographic studies reporting late-term reductions in placentome size (9, 11) and with the well-documented decline in oPL secretion nearing parturition (15). These late-term shifts likely reflect physiological transitions associated with final fetal maturation and endocrine adjustments preparing the ewe for parturition and lactation. Experimental work by Leibovich et al. (20) demonstrated that altering oPL availability does not impair reproductive success but can influence fetal growth and mammary development, indicating that oPL participates in broader periparturient adaptation. Although such experimental frameworks differ from the present observational design, they underscore that oPL contributes to late gestational adaptation through its coordinated effects on fetal growth, maternal metabolic regulation, and mammary gland development, thereby providing a physiological context for the strengthened structural–endocrine coupling observed in the present study.

Regarding the study’s methodological framework, the use of a homogeneous flock of multiparous Kivircik ewes with comparable body condition scores, the restriction to singleton pregnancies, and the consistent ultrasonographic protocol minimised extraneous variability and strengthened the reliability of serial measurements. These design choices are aligned with recommendations from previous ultrasonographic evaluations in sheep (8, 9, 21). Nonetheless, intrinsic biological variability in placentome architecture likely contributes to the early-gestation dispersion observed in both parameters.

The present findings indicate that placentome diameter and maternal oPL concentration each follow distinct yet interrelated developmental trajectories throughout gestation. Their relationship is not static but evolves in a stage-dependent manner, with the strongest coupling emerging in late pregnancy when structural and endocrine components appear most functionally aligned. The use of a homogeneous flock of multiparous Kivircik ewes, restriction to singleton pregnancies, and a consistent ultrasonographic protocol alongside oPL analyses provided a controlled framework for examining these temporal patterns. Within these methodological boundaries, the observed results contribute to a more integrated interpretation of placental structure and endocrine function during ovine gestation. At the same time, placentome diameter represents a simplified structural indicator and may not fully reflect changes in placentome shape, thickness, or internal vascular architecture, particularly during late gestation when placental remodelling predominates. Accordingly, future studies incorporating placentome classification, vascular parameters, or detailed cotyledonary morphometrics in combination with endocrine profiling may further refine the understanding of structural–functional relationships and extend their relevance for reproductive research and management.

## Data Availability

The original contributions presented in the study are included in the article/supplementary material, further inquiries can be directed to the corresponding author.
